# Population Behavior Changes Underlying Phasic Shifts of SARS-CoV-2 Exposure Settings Across 3 Omicron Epidemic Waves in Hong Kong: Prospective Cohort Study

**DOI:** 10.2196/51498

**Published:** 2024-06-19

**Authors:** Chin Pok Chan, Shui Shan Lee, Tsz Ho Kwan, Samuel Yeung Shan Wong, Eng-Kiong Yeoh, Ngai Sze Wong

**Affiliations:** 1 JC School of Public Health and Primary Care Faculty of Medicine The Chinese University of Hong Kong Hong Kong China; 2 Stanley Ho Centre for Emerging Infectious Diseases Faculty of Medicine The Chinese University of Hong Kong Hong Kong China; 3 S.H. Ho Research Centre for Infectious Diseases Faculty of Medicine The Chinese University of Hong Kong Hong Kong China; 4 Centre for Health Systems and Policy Research Faculty of Medicine The Chinese University of Hong Kong Hong Kong China

**Keywords:** exposure risk, contact setting, social distancing, epidemic control, participatory surveillance, SARS-CoV-2, COVID-19

## Abstract

**Background:**

Exposure risk was shown to have affected individual susceptibility and the epidemic spread of COVID-19. The dynamics of risk by and across exposure settings alongside the variations following the implementation of social distancing interventions are understudied.

**Objective:**

This study aims to examine the population’s trajectory of exposure risk in different settings and its association with SARS-CoV-2 infection across 3 consecutive Omicron epidemic waves in Hong Kong.

**Methods:**

From March to June 2022, invitation letters were posted to 41,132 randomly selected residential addresses for the recruitment of households into a prospective population cohort. Through web-based monthly surveys coupled with email reminders, a representative from each enrolled household self-reported incidents of SARS-CoV-2 infections, COVID-19 vaccination uptake, their activity pattern in the workplace, and daily and social settings in the preceding month. As a proxy of their exposure risk, the reported activity trend in each setting was differentiated into trajectories based on latent class growth analyses. The associations of different trajectories of SARS-CoV-2 infection overall and by Omicron wave (wave 1: February-April; wave 2: May-September; wave 3: October-December) in 2022 were evaluated by using Cox proportional hazards models and Kaplan-Meier analysis.

**Results:**

In total, 33,501 monthly responses in the observation period of February-December 2022 were collected from 5321 individuals, with 41.7% (2221/5321) being male and a median age of 46 (IQR 34-57) years. Against an expanding COVID-19 vaccination coverage from 81.9% to 95.9% for 2 doses and 20% to 77.7% for 3 doses, the cumulative incidence of SARS-CoV-2 infection escalated from <0.2% to 25.3%, 32.4%, and 43.8% by the end of waves 1, 2, and 3, respectively. Throughout February-December 2022, 52.2% (647/1240) of participants had worked regularly on-site, 28.7% (356/1240) worked remotely, and 19.1% (237/1240) showed an assorted pattern. For daily and social settings, 4 and 5 trajectories were identified, respectively, with 11.5% (142/1240) and 14.6% (181/1240) of the participants gauged to have a high exposure risk. Compared to remote working, working regularly on-site (adjusted hazard ratio [aHR] 1.47, 95% CI 1.19-1.80) and living in a larger household (aHR 1.12, 95% CI 1.06-1.18) were associated with a higher risk of SARS-CoV-2 infection in wave 1. Those from the highest daily exposure risk trajectory (aHR 1.46, 95% CI 1.07-2.00) and the second highest social exposure risk trajectory (aHR 1.52, 95% CI 1.18-1.97) were also at an increased risk of infection in waves 2 and 3, respectively, relative to the lowest risk trajectory.

**Conclusions:**

In an infection-naive population, SARS-CoV-2 transmission was predominantly initiated at the workplace, accelerated in the household, and perpetuated in the daily and social environments, as stringent restrictions were scaled down. These patterns highlight the phasic shift of exposure settings, which is important for informing the effective calibration of targeted social distancing measures as an alternative to lockdown.

## Introduction

In 2020-2021, Hong Kong’s COVID-19 burden in the population was among the lowest in the Asia Pacific region (<1%). Ranging from border control and mandatory mask wearing to social activity restrictions, the implementation of various nonpharmaceutical interventions (NPIs) contributed to the successful control initially. It was not until February 2022 when the SARS-CoV-2 Omicron outbreak swept through the city and caused over 2.6 million cases (35%) in a 7.4 million population by the end of the year [1]. Albeit the epidemic becoming more controllable today, it is still unclear what factors had contributed to the initial surge of Omicron and subsequent waves of infection. Since the effectiveness of vaccinations against emerging variants is waning, investigations into the impact of the population’s behavior on virus transmission would therefore be crucial [2].

Exposure risk and its association with SARS-CoV-2 infection have been examined from various perspectives. Previous studies have analyzed the social contact and mobility pattern to model the change in the transmission dynamics [3,4]. However, the application of these metrics as a proxy for aggregated exposure risks has ignored the heterogeneity of the exposure profiles across the population [5]. The distribution of population activities in various community settings is often overlooked. Former research has presented a remarkable difference in the infection rate following SARS-CoV-2 exposure to a household and health care source [6,7]. Thus, the nature of social contacts in different settings could also play an important role in affecting the level of virus exposure and infection risk. Based on contact-tracing data, the transmission dynamics of SARS-CoV-2 can also be observed to be largely variable among clusters of infection emerging from different environments [8-10]. Yet, evidence formally appraising the association between exposure and outbreaks in a broader community context remains scarce.

As an integral part of NPIs, different combinations of social distancing interventions were implemented worldwide to tackle the COVID-19 pandemic. The use of regulatory restriction was shown to have effectively blocked transmission chains associated with social events [11], while prohibiting dine-in services and imposing a work from home (WFH) order were deemed to have rapidly brought down the scale of virus spread [12]. A multi-country analysis in 2020-2022 also revealed the fluctuation in social contact patterns in different settings during the pandemic and highlighted its connection with different containment measures [13]. The above evidence conjectured that the whereabouts and dynamics of virus exposure could be influenced by the coverage and stringency of social distancing measures [14]. Yet, their interplay was still vaguely understood as the epidemic progressed through waves driven by different SARS-CoV-2 variants in the real world.

In recent decades, participatory surveillance has been increasingly used as a complementary tool to detect exposure and disease patterns in community outbreaks [15,16]. Without imposing a complete lockdown, the comparison between infected and uninfected individuals in their activity patterns by setting would enable the identification of the changing hot spots for SARS-CoV-2 exposure as the epidemic unfolds. Against these backgrounds, this study aims to examine the dynamics of population exposure risks in different settings and to evaluate its association with SARS-CoV-2 infection across the 3 Omicron epidemic waves in 2022 in Hong Kong.

## Methods

### Participant Recruitment and Study Design

In the form of a prospective cohort, an ongoing web-based participatory surveillance platform was set up in Hong Kong with territory-wide recruitment in March-June 2022. Taking individual households as a sampling unit, cluster sampling was performed based on a sampling frame called “building groups”—a demarcation system codeveloped by the Census and Statistics Department of the Hong Kong Government and the Centamap Company Limited for grouping local households with a similar socioeconomic background for census data analysis. The demarcation system, updated as of 2016, with 3083 defined building groups (strata) covering 165,965 residential buildings, was used as the sampling frame of this study. In each stratum resided by at least 1000 individuals, 12-14 households were randomly selected to receive an invitation letter bearing a unique code mapped to their residential address. A representative household member aged 18 years or older, residing in Hong Kong, and who understood Chinese or English was invited to participate. Following registration on the platform by using the unique household code, participants who consented were asked to fill in a baseline questionnaire followed by monthly updates during the follow-up period.

### Ethics Approval

Ethics approval was sought from the Survey and Behavioral Research Ethics Committee of the Chinese University of Hong Kong (SBRE-21-0048). Informed e-consent was obtained from each participant at the time of registration. The survey data set was deidentified by removing participants’ residential addresses. Separately, residential addresses used for recruitment and incentive delivery were stored in another data set with matched study IDs to preserve anonymity. All data sets were password-protected and only accessed by designated research staff and investigators. Upon completion of the baseline questionnaire, participants were entitled to a HK $50 cash voucher (HK $7.8=US $1). During follow-up, additional HK $50 vouchers would be offered to participants who had completed at least 3 monthly updates every 6 months.

### Survey Instrument

Available in both Chinese and English, the baseline questionnaire could be completed in 20 minutes and covered the participants’ particulars on (1) sociodemographic characteristics; (2) household size; (3) chronic illness status; (4) baseline history of SARS-CoV-2 infection and COVID-19 vaccination; (5) frequency of rapid antigen/nucleic acid testing for SARS-CoV-2 in the previous month; (6) monthly pattern of work, daily, and social activities; (7) nature of workplace (health care/non–health care) and the number of workplace contacts; (8) perceived risk of SARS-CoV-2 exposure (on an 11-point Likert scale from 0 to 10); and (9) suspected presence of infected individuals among daily contacts (yes/no). For the ensuing monthly updates, participants were asked to self-report any COVID-19 vaccine uptake and incident of SARS-CoV-2 infection, defined by a positive result from a polymerase chain reaction or rapid antigen test regardless of the presence of symptoms. Updates on sections 5-9 and household infection status based on the situation in the previous month were also requested. A personalized survey link was sent to each participant by email on the first of each month and made accessible for 14 days. Paper questionnaires were supplied upon request (n=23).

### Definition of Exposure Risk and Tiers of Social Distancing Policy

In this study, exposure risk was defined as the intensity of behavior leading to potential virus exposure, while its dynamics were examined in 4 contexts: household, workplace, social setting, and daily setting. Persons living in larger households and working entirely outside the home in a month were expected to have a greater exposure risk. For daily and social settings, the exposure risk was measured by their frequency of engagement in different types of daily, leisure, and social activities. Details of the exposure risk definition are illustrated in Table 1. Based on the stringency of the regulations imposed on the operations of catering businesses, bars/pubs, and a range of high-risk premises and activities; restrictions on social gatherings; and government recommendations on WFH practices, the evolvement of the local social distancing policies was divided into 4 tiers. The details of the regulations together with other NPIs are delineated in Figure S1 in Multimedia Appendix 1.

**Table 1:** Study definitions of exposure risk in different settings.

Exposure setting	Proxy question for exposure risk	Measurements used in analyses	Assumption on exposure risk
Household	Number of coliving members in the household at baseline	Continuous variable: household size/ordinal variable with 4 levels: 1 person (living alone) 2 persons 3-4 persons ≥5 persons	Constant throughout due to repeated contact with family members; higher exposure risk in larger families
Workplace	Reports on whether one had practiced WFH^a^ completely, intermittently, or worked entirely outside home in each month	Ordinal variable with 3 levels: Completely WFH/not working Intermittently WFH Work entirely outside home	Varied; higher exposure risk in those who worked outside the home for the entire month
Daily	Daily activity^b^: reported number of days one had (1) visited an eatery, (2) visited a shopping mall/market, and (3) used public transportation last week in each month (scale of 1: never; 2: 1 day; 3: 2-3 days; 4: 4-6 days; 5: everyday) Leisure activity: reports on whether one had ever visited sauna/bathhouse, massage/beauty parlor, fitness center, beach/pool, sport facility, or entertainment venue such as a cinema in each month	Ordinal variable with 4 levels: Inactive and without leisure activity Inactive and with leisure activity Active and without leisure activity Active and with leisure activity	Varied; higher exposure risk in those who had a more active daily lifestyle and participated in leisure activity
Social	Reported number of times one had paid visit to relatives/friends’ home or vice versa, dined out for the purpose of gathering, gone to bar/club, karaoke room, party room, attended banquet, gone on a local hotel vacation, and outdoor travel (eg, hiking/camping) in each month	Ordinal variable with 4 levels: None 1-3 times 4-7 times ≥8 times	Varied; higher exposure risk in those who had attended more social events

^a^WFH: work from home.

^b^The frequencies for each of the daily activities were standardized and averaged for each participant. Those with a computed average higher than the population median were classified as active or otherwise inactive for a particular month.

### Statistical Analysis

Participants’ baseline characteristics were summarized using descriptive statistics. The geographical representativeness of the recruited households was then assessed by comparison with the census distribution [17]. The cumulative incidence of SARS-CoV-2 infection and monthly proportion of participants reporting an infection were profiled against the number documented by the government, while age and sex adjustments were performed in reference to the population demographics [17]. The patterns of age-specific COVID-19 burden and vaccination coverage were also illustrated.

Based on the reported work pattern and computed exposure risk level in daily and social settings, the temporal dynamics of each were differentiated using a latent class growth model—a mixture model for identifying underlying subgroups exhibiting similar growth trajectories [18]. Only participants with a missing response in at most 1 month during each epidemic wave were included in the analysis. Individual responses of specific months, during which SARS-CoV-2 infection was reported in themselves or their household, were also eliminated so that the biased activity level resulting from isolation/quarantine was not considered. A 2-stage optimization of maximum likelihood was adopted to model the intercept, linear, and quadratic growth factor, with an increasing number of latent classes from 1 to 7. Goodness of fit was indicated by the lowest Bayesian information criterion (BIC) and an entropy of >0.8. Elbow plot was used in cases when the stated criteria were still met in the 7-class model. Based on the selected models, the class-specific exposure risk trajectories were plotted on a probability scale. Factors associated with each trajectory were determined by a multinomial mixed effects model.

The time patterns of SARS-CoV-2 infection among participants with different household sizes, work patterns, and daily and social exposure risk trajectories were examined using Kaplan-Meier analysis, while the difference in the infection rate in each epidemic wave was compared using relative risk (RR). Their relationships with SARS-CoV-2 infection risk were then parameterized using Cox proportional hazards models adjusted for sociodemographic characteristics and vaccination status before the Omicron outbreak. Subgroup analysis was also conducted between the subset of participants with different pre-Omicron vaccination statuses. R software (version 4.1.2; R Foundation for Statistical Computing) and Mplus (version 8.8; Muthen & Muthen) were used to perform statistical analyses. All tests were 2-tailed, and the significance was denoted by *P*<.05.

## Results

### Baseline Characteristics

Between March and June 2022, representative participants from 5321 households were recruited out of 41,132 invitations (response rate 12.9%). Concerning the observation period from February to December 2022, over 3000 survey entries were received each month except for the first 2 months of recruitment, amounting to a total of 33,501 responses. Two-thirds (3553/5321, 66.8%) of the recruited participants completed at least 50% of the monthly surveys, with 58.3% (3100/5321) being female, and the median age was 46 (IQR 34-57) years. A majority of the participants were in full-time employment or self-employed (3208/5321, 60.3%) and had attained tertiary education or above (3325/5321, 62.5%) (Table 2). The recruited households had a mean size of 3.1 (SD 1.5) persons, and 11.5% (607/5321) were single-person households. The geographical distribution of the recruited households was within a 1% difference compared to the census data for all 18 districts in the territory of Hong Kong (Table S1 in Multimedia Appendix 1).

**Table 2:** Baseline characteristics of the participants (N=5321).

Characteristics		Values, n (%)
**Sex, n (%)**		
	Male	2221 (41.7)
	Female	3100 (58.3)
**Age (years)**		
	Median (IQR)	46 (34-57)
	18-29, n (%)	732 (13.8)
	30-39, n (%)	1188 (22.3)
	40-49, n (%)	1180 (22.2)
	50-64, n (%)	1614 (30.3)
	≥65, n (%)	607 (11.4)
**Ethnicity, n (%)**		
	Local (Hong Kong) Chinese	5190 (97.5)
	Nonlocal Chinese/other ethnicities	131 (2.5)
**Education level, n (%)**		
	Primary education or below	175 (3.3)
	Secondary education	1821 (34.2)
	Diploma/associate degree	803 (15.1)
	Bachelor’s degree or above	2522 (47.4)
**Employment status, n (%)**		
	Full-time employment/self-employed	3208 (60.3)
	Part-time/temporary employment	288 (5.4)
	Student	251 (4.7)
	Homemaker	406 (7.6)
	Unemployed	350 (6.6)
	Retired	816 (15.3)
**Monthly income^a^ range (n=3739; missing: n=8), n (%)**		
	<HK $10,000	434 (11.6)
	HK $10,000-19,999	895 (23.9)
	HK $20,000-29,999	759 (20.3)
	HK $30,000-59,999	972 (26)
	≥HK $60,000	410 (11)
	Refuse to answer	269 (7.2)
**Reported chronic illness^b^ (unsure: n=246), n (%)**		
	No	3638 (71.7)
	Yes	1437 (28.3)
**Household size (missing: n=62)**		
	Mean (SD)	3.1 (1.5)
	1 (living alone), n (%)	607 (11.5)
	2 persons, n (%)	1478 (28.1)
	3-4 persons, n (%)	2264 (43.1)
	5-6 persons, n (%)	805 (15.3)
	≥7 persons, n (%)	105 (2)

^a^HK $7.8=US $1.

^b^Including any diagnosed conditions that require long-term clinical follow-up or medications, such as hypertension, diabetes mellitus, stroke, and asthma.

### SARS-CoV-2 Burden and COVID-19 Vaccination Uptake

**Figure 1:**
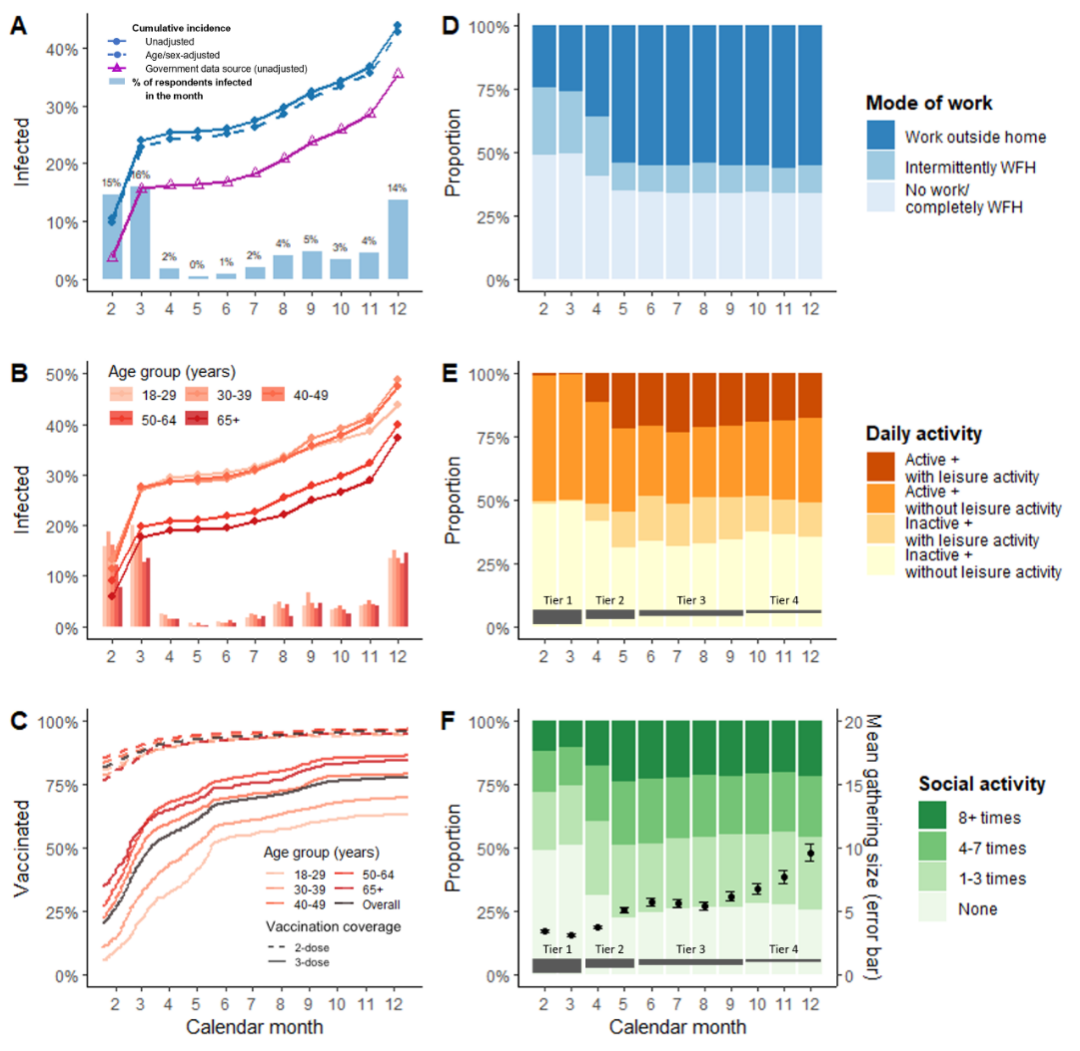
Temporal pattern of SARS-CoV-2 infection, COVID-19 vaccination coverage, mode of work, and activity pattern in daily and social settings from February to December 2022. (A) The bar graph represents the monthly proportion of respondents reporting an incident of SARS-CoV-2 infection, while the lines represent the cumulative incidence based on the cumulative number of participants reporting SARS-CoV-2 infection reported up to a particular month divided by the total number of cohort participants (N=5321). (B) The age-specific pattern is shown. (C) Vaccination coverage by 2 and 3 doses and age group. (D-F) Mode of work categorized into no work/completely work from home, intermittently work from home, and work outside home. For daily setting, the weekly frequency of patronizing eateries and malls/markets, and using public transportation was assessed. Participants with a mean standardized frequency of 3 items above the median were considered to be active or otherwise inactive. Leisure activity refers to visits to any of the following venues in a particular month: sauna/bathhouse, massage/beauty parlor, fitness center, beach/swimming pool, sports facility, and entertainment venues such as cinema. For social settings, the number of times participants attenended social activities, including visiting relatives/friends’ homes or vice versa, dining out for the purpose of gathering, going to a bar/club, karaoke room, party room, banquet, hotel vacation, and domestic travel, were assessed and categorized into none, 1-3 times, 4-7 times, and ≥8 times each month. The error bar represents the population average of the mean gathering size of all social activities reported by an individual in each month and the 95% CI. Detailed in Multimedia Appendix 1 (Figure S1), the 4 tiers in (D) and (E) indicate the stringency level of the effective social distancing policy (tier 1: most stringent). WFH: work from home.

As of December 2022, a total of 2332 SARS-CoV-2 infections were reported, adding up to a cumulative incidence of 43.8% since 2020 (Figure 1). The age- and sex-adjusted estimate was 42.8%, which was 7% higher than the government figure. The number of incident infections peaked in March 2022 (273/1718, 15.9%), reached the trough in May (13/3505, 0.4%), rebounded in August (127/3208, 3.9%), and surged in December (410/3206, 13.5%), giving rise to 3 Omicron waves in 2022 (wave 1: February to April, wave 2: May to September, and wave 3: October to December). Overall, hospitalization was required in 20 of 2332 cases (0.9%), while reinfection with at least 3 months from the previous infection episode was reported in 45 (1.9%) participants. Stratified by age group, individuals aged 30-39 years had the highest cumulative incidence of 48.8% (580/1188), while the population aged ≥65 years was least affected by the epidemic (226/607, 37.2%). Over 80% (4231/5167) and 20% (1043/5208) of the participants had received at least 2 and 3 doses of COVID-19 vaccine before February 2022, respectively. Correspondingly, the 2 coverages escalated to 95.9% (4954/5167) and 77.7% (4047/5208) at the end of the year after the administration of vaccine passes requiring 2 and 3 doses by May and June (Figure S1 in Multimedia Appendix 1). A noticeably slower third dose uptake was observed throughout in younger age groups.

### Activity Pattern in Workplace, Daily Settings, and Social Settings

When the Omicron outbreak first broke out in February 2022, the most stringent tier 1 social distancing measures were in place. In reference to the government advice, 26.8% (656/2444) and 49% (1198/2444) reported intermittent and complete WFH practice (or not doing any work), while one-quarter of the participants (590/2444, 24.1%) continued to work on-site in their workplace. Due to the mass closure of community facilities, almost none had reported participation in leisure activities in daily settings. Half of the participants (1202/2444, 49.2%) refrained from taking part in any social activity, whereas 22.7% (555/2444), 16% (392/2444), and 12.1% (295/2444) still went out for 1-3 times, 4-7 times, and ≥8 times, respectively, despite the restrictions, with a mean gathering size of 3.4 persons. Following the tier 2 relaxation in May 2022, the proportion working on-site returned to 54.1% (1899/3505), with 11% (384/3505) maintaining their intermittent WFH practice. Based on their reported frequency of visiting eateries and shopping malls/markets, and using public transportation, living an active lifestyle with engagement in leisure activity was acknowledged in 21.8% (763/3505) of the participants, compared to 31% (1087/3505) of the participants who were considered to be inactive. Social activity was also restored, with 24% (839/3502) of the participants reporting engagement for ≥8 times per month in May. The overall pattern of work, daily, and social activities remained stable, as social distancing measures continued to ease, while the average gathering size increased from 5.1 in May to 9.6 persons in December 2022.

### Trajectories of Work Pattern, Daily, and Social Exposure Risk and the Associated Characteristics

Excluding those with inadequate follow-up responses amid the 3 waves, a total of 1240 participants were included in latent class growth analyses. This subgroup was similar to the rest, except that slightly more of those being excluded were local (Hong Kong) Chinese (1222/1240, 98.5% vs 3968/4081, 97.2%; *P*=.01) with a higher median age (48 in those included vs 45 years in those excluded from the latent growth class analyses; *P*<.001; Table S2 in Multimedia Appendix 1).

**Figure 2:**
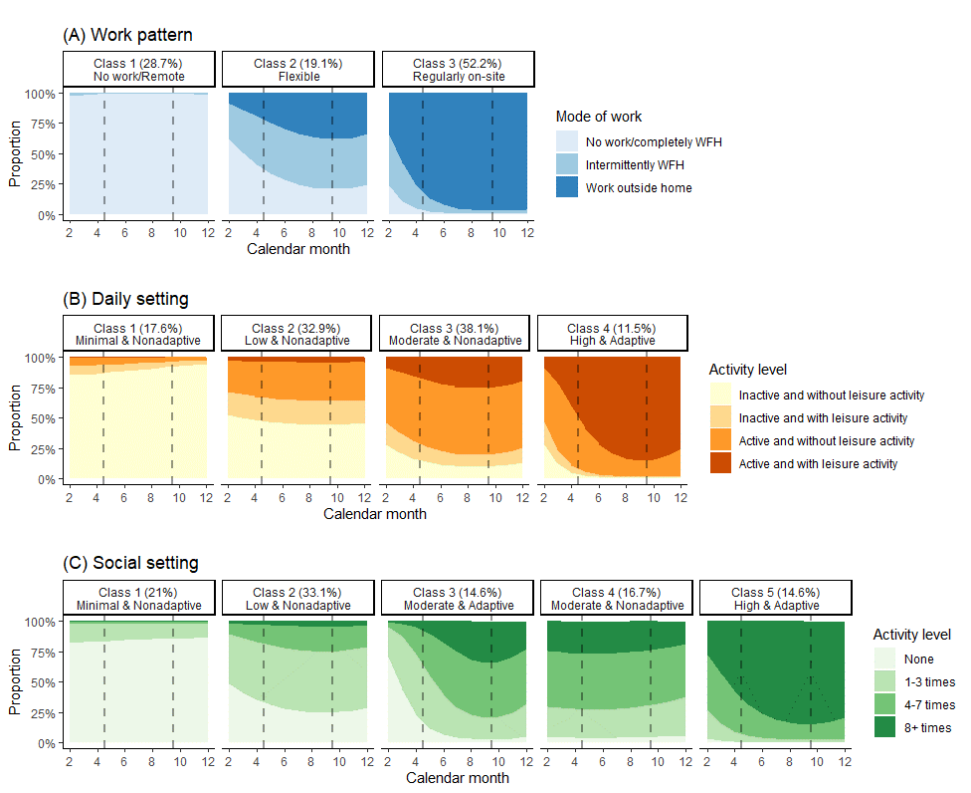
Trajectories of the work pattern and exposure risk in daily and social settings by latent class growth models. Compartments divided by the dashed line indicate the 3 periods of Omicron waves in 2022: wave 1 (February to April), wave 2 (May to September), and wave 3 (October to December).

To differentiate between work patterns, the best fit was demonstrated in the 3-class latent class growth model (BIC 12,308.19, entropy 0.957; Table S3 and Figure S2 in Multimedia Appendix 1). Approximately 28.7% (356/1240) of the participants were not working or had worked remotely throughout the year (class 1), while about half (class 3: 647/1240, 52.2%) regularly worked outside the home, with 19.1% (237/1240) following an assorted work pattern (class 2; Figure 2).

To illustrate the daily exposure risk dynamics, the 4-class model showed the best fit (BIC 25,712.03, entropy 0.801). With differing levels of exposure risk and behavioral responsiveness to the epidemic, 17.6% (218/1240), 32.9% (408/1240), 38.1% (472/1240), and 11.5% (142/1240) were classified as “class 1: minimal and nonadaptive,” “class 2: low and nonadaptive,” “class 3: moderate and nonadaptive,” and “class 4: high and adaptive,” respectively. Compared to class 1, the other classes were significantly more likely to be in full-time employment (class 2: adjusted odds ratio [aOR] 2.05, 95% CI 1.13-3.73; class 3: aOR 3.22, 95% CI 1.76-5.89; class 4: aOR 2.81, 95% CI 1.48-5.37) and self-perceive a higher chance of virus exposure (class 2: aOR 1.18, 95% CI 1.06-1.30; class 3: aOR 1.21, 95% CI 1.09-1.34; class 4: aOR 1.31, 95% CI 1.16-1.47; Table 3). Significantly more class 4 participants had received tertiary education or above (aOR 2.28, 95% CI 1.18-4.39) and undergone SARS-CoV-2 testing more frequently (aOR 1.03, 95% CI 1.00-1.06).

**Table 3:** Multinomial mixed effects model on factors associated with daily exposure risk trajectories.

Variables			Class 1 (minimal and nonadaptive)		Class 2 (low and nonadaptive), aOR^a^ (95% CI)		Class 3 (moderate and nonadaptive), aOR (95% CI)		Class 4 (high and adaptive), aOR (95% CI)
**Sex**									
	Male	Reference		1.00		1.00		1.00	
	Female	Reference		0.93 (0.53-1.61)		1.81 (1.03-3.17)*		1.94 (1.08-3.46)*	
Age (years)			Reference		1.01 (0.99-1.04)		1.03 (1.01-1.06)**		1.06 (1.03-1.09)***
**Education level**									
	Secondary education or below	Reference		1.00		1.00		1.00	
	Tertiary education or above	Reference		1.09 (0.59-2.02)		0.95 (0.51-1.77)		2.28 (1.18-4.39)*	
Full-time worker			Reference		2.05 (1.13-3.73)*		3.22 (1.76-5.89)***		2.81 (1.48-5.37)**
Household size			Reference		1.07 (0.88-1.31)		1.11 (0.91-1.35)		1.08 (0.88-1.33)
Reported chronic illness			Reference		1.08 (0.56-2.07)		0.93 (0.48-1.79)		1.47 (0.76-2.87)
Monthly frequency of SARS-CoV-2 testing^b^			Reference		1.01 (0.99-1.04)		1.03 (1.00-1.05)		1.03 (1.00-1.06)*
Suspected SARS-CoV-2 infection among daily contacts^b^			Reference		1.12 (0.77-1.62)		1.17 (0.81-1.70)		1.38 (0.91-2.08)
Perceived risk of SARS-CoV-2 exposure (range 0-10)^b^			Reference		1.18 (1.06-1.30)**		1.21 (1.09-1.34)***		1.31 (1.16-1.47)***

^a^aOR: adjusted odds ratio.

^b^Time-varying predictor with the participant set to be the random effect.

*P<.05; **P<.01; ***P<.001.

For social exposure risk, the best model fit was observed in the 5-class model (BIC 25,503.3, entropy 0.816). Most participants (411/1240, 33.1%) belonged to the “class 2: low and nonadaptive” group, followed by “class 1: minimal and nonadaptive” (260/1240, 21%), “class 3: moderate and adaptive” (181/1240, 14.6%), “class 4: moderate and nonadaptive” (207/1240, 16.7%), and “class 5: high and adaptive” (181/1240, 14.6%). Participants with a higher social exposure risk were in general older (class 2: aOR 1.04, 95% CI 1.01-1.06; class 3: aOR 1.05, 95% CI 1.02-1.07; class 4: aOR 1.07, 95% CI 1.04-1.09; class 5: aOR 1.06, 95% CI 1.04-1.09) and more educated (class 3: aOR 3.70, 95% CI 1.95-7.01; class 4: aOR 4.57, 95% CI 2.35-8.73; class 5: aOR 5.42, 95% CI 2.81-10.46; Table 4). Female behaviors appeared to be more responsive to adjustments in social distancing policy (class 3: aOR 2.30, 95% CI 1.30-4.09; class 5: aOR 2.09, 95% CI 1.17-3.74).

**Table 4:** Multinomial mixed effects model on factors associated with social exposure risk trajectories.

Variables			Class 1 (minimal and nonadaptive)		Class 2 (low and nonadaptive), aOR^a^ (95% CI)		Class 3 (moderate and adaptive), aOR (95% CI)		Class 4 (moderate and nonadaptive), aOR (95% CI)		Class 5 (high and adaptive), aOR (95% CI)
**Sex**											
	Male	Reference		1.00		1.00		1.00		1.00	
	Female	Reference		1.69 (0.96-2.99)		2.30 (1.30-4.09)**		1.67 (0.94-2.98)		2.09 (1.17-3.74)*	
Age (years)			Reference		1.04 (1.01-1.06)**		1.05 (1.02-1.07)***		1.07 (1.04-1.09)***		1.06 (1.04-1.09)***
**Education level**											
	Secondary education or below	Reference		1.00		1.00		1.00		1.00	
	Tertiary education or above	Reference		1.69 (0.90-3.19)		3.70 (1.95-7.01)***		4.57 (2.39-8.73)***		5.42 (2.81-10.46)***	
Full-time worker			Reference		1.75 (0.94-3.25)		0.99 (0.54-1.83)		1.41 (0.75-2.65)		0.67 (0.36-1.25)
Household size			Reference		1.04 (0.85-1.28)		1.13 (0.92-1.38)		0.95 (0.78-1.17)		0.92 (0.75-1.13)
Reported chronic illness			Reference		0.77 (0.39-1.52)		1.20 (0.62-2.34)		1.06 (0.54-2.07)		0.74 (0.38-1.44)
Monthly frequency of SARS-CoV-2 testing^b^			Reference		1.00 (0.97-1.02)		1.00 (0.97-1.03)		0.99 (0.97-1.02)		1.00 (0.97-1.03)
Suspected SARS-CoV-2 infection among daily contacts^b^			Reference		1.23 (0.85-1.78)		1.41 (0.94-2.10)		1.38 (0.93-2.04)		1.59 (1.06-2.37)*
Perceived risk of SARS-CoV-2 exposure (range 0-10)^b^			Reference		1.02 (0.92-1.14)		1.07 (0.96-1.20)		1.07 (0.96-1.20)		1.08 (0.96-1.21)

^a^aOR: adjusted odds ratio.

^b^Time-varying predictor with the participant set to be the random effect.

*P<.05; **P<.01; ***P<.001.

### Timing and Risk Factors of SARS-CoV-2 Infection

**Figure 3:**
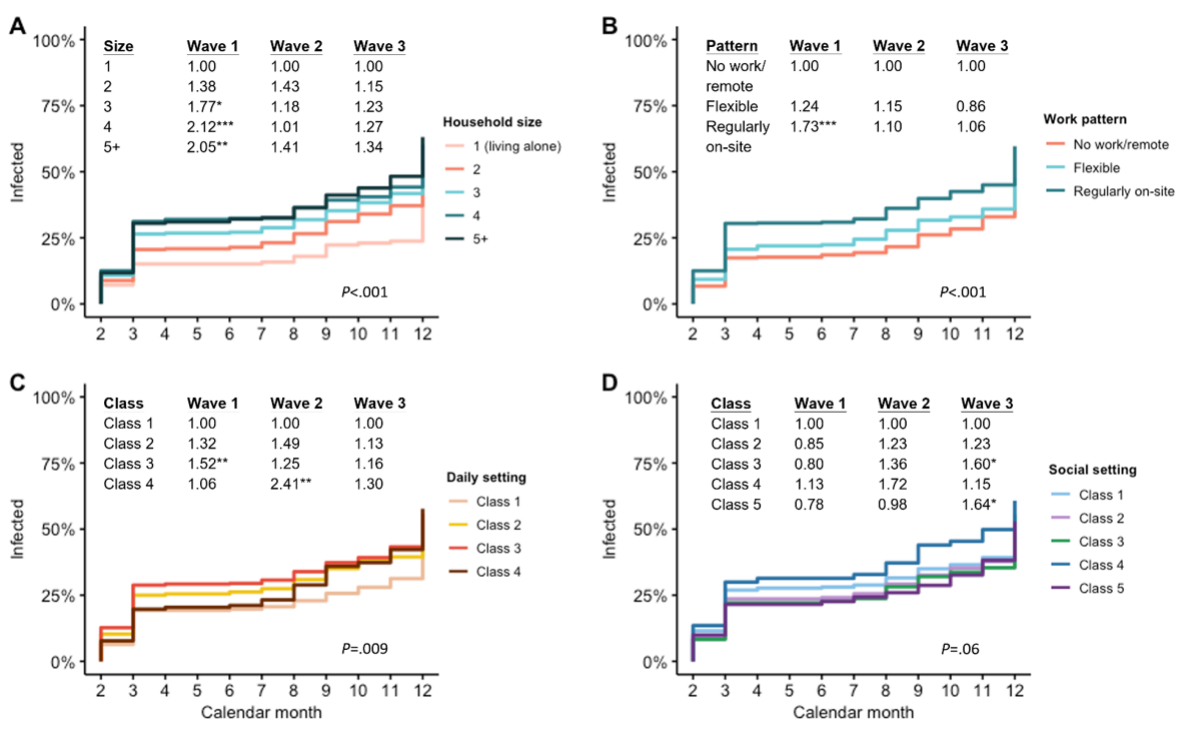
Results of Kaplan-Meier analysis on the timing of the SARS-CoV-2 infection among groups of varying household size, work pattern, daily, and social exposure risk trajectories. An event was defined by an incident of SARS-CoV-2 infection. Relative risks based on the comparison of SARS-CoV-2 infection rates were computed among different groups at each Omicron wave (wave 1: February to April; wave 2: May to September; and wave 3: October to December) in 2022. The significance value of the log rank test is also displayed in each panel. *P<.05, **P<.01,***P<.001.

Overall, there was a significant heterogeneity in SARS-CoV-2 infection risk among groups of varying household size (*χ*^2^_4_=28.6; *P*<.001), work pattern (*χ*^2^_3_=25.6; *P*<.001), and daily exposure risk trajectories (*χ*^2^_3_=11.6; *P*=.009; Figure 3). When the results were adjusted for sex, age, education, work pattern, and pre-Omicron COVID-19 vaccination status, participants living in a larger household (adjusted hazard ratio [aHR] 1.12, 95% CI 1.08-1.20) and being a health care worker (aHR 1.38, 95% CI 1.29-2.26) were linked to a significantly higher infection risk (Table 5). Those who worked regularly on-site (aHR 1.47, 95% CI 1.19-1.80) and had more workplace contacts (hazard ratio [HR] 1.01, 95% CI 1.00-1.02) with a higher daily (class 2: aHR 1.38, 95% CI 1.07-1.77; class 3: aHR 1.49, 95% CI 1.16-1.91; class 4: aHR 1.46, 95% CI 1.07-2.00) and social exposure risk (class 4: aHR 1.52, 95% CI 1.18-1.97) were also more prone to SARS-CoV-2 infection. Stratified by the epidemic wave, individuals living in a household with size of 3 (RR 1.77; *P*=.01), 4 (RR 2.12; *P*<.001), and ≥5 persons (RR 2.05; *P*=.002) had a higher risk of infection in wave 1 compared to those living alone. A similar association was also identified in those who regularly worked outside the home (RR 1.73; *P*<.001) and daily exposure risk trajectory class 3 participants (RR 1.52; *P*=.008). Significantly more daily exposure risk trajectory class 4 participants had acquired SARS-CoV-2 infection in wave 2 (RR 2.41; *P*=.007). In contrast, an excess of social exposure risk trajectory class 3 (RR 1.60; *P*=.03) and class 5 (RR 1.64; *P*=.02) participants became infected in wave 3.

Stratified by the COVID-19 vaccination status, a higher cumulative incidence was reported in participants who had received 0-2 doses before the Omicron outbreak (515/946, 54.4% vs 112/262, 42.7%). In this subgroup, household, workplace, daily, and social exposure risk remained significantly associated with an episode of SARS-CoV-2 infection. However, for the rest who had received 3 doses or more, only larger household size (HR 1.13, 95% CI 1.01-1.28) and regular on-site work pattern (HR 1.56, 95% CI 1.03-1.36) were significant risk factors in the unadjusted model. The effect of daily exposure risk was, however, absent.

**Table 5:** Cox proportional-hazards model on the risk of SARS-CoV-2 infection and subgroup analyses.

Variables		Overall (n=1240)^a^			Pre-Omicron vaccination status: 0-2 doses (n=946)^b^			Pre-Omicron vaccination status: ≥3 doses (n=262)^b^	
		HR^c^ (95% CI)	aHR^d^ (95% CI)	HR (95% CI)		aHR (95% CI)	HR (95% CI)		aHR (95% CI)
**Sex**									
	Male	1.00	—^e^	1.00		—	1.00		—
	Female	1.10 (0.94-1.29)	—	1.19 (1.00-1.43)		—	0.81 (0.56-1.18)		—
Age (in years)		0.99 (0.98-0.99)***	—	0.99 (0.99-1.00)**		—	0.98 (0.97-1.00)*		—
**Education level**									
	Secondary education or below	1.00	—	1.00		—	1.00		—
	Tertiary education or above	1.06 (0.90-1.25)	—	1.10 (0.91-1.32)		—	0.90 (0.62-1.31)		—
Health care worker		1.70 (1.29-2.26)***	1.38 (1.03-1.86)*	1.74 (1.26-2.40)***		1.46 (1.05-2.03)*	1.43 (0.75-2.75)		1.28 (0.65-2.52)
Number of workplace contacts in February 2022 (n=577)		1.01 (1.00-1.02)**	—	1.01 (1.00-1.02)*		—	1.01 (0.96-1.07)		—
Household size (missing=22)		1.14 (1.08-1.20)***	1.12 (1.06-1.18)***	1.13 (1.07-1.20)***		1.11 (1.05-1.18)***	1.13 (1.01-1.28)*		1.10 (0.98-1.25)
**COVID-19 vaccination status prior to Omicron outbreak (missing=32)**									
	Unvaccinated/1 dose	1.00	—	—		—	—		—
	2 doses	0.91 (0.74-1.13)	—	—		—	—		—
	≥3 doses	0.65 (0.49-0.84)**	—	—		—	—		—
**Work pattern**									
	No work/remote	1.00	1.00	1.00		1.00	1.00		1.00
	Flexible	1.13 (0.88-1.44)	1.01^f^ (0.78-1.31)	1.10 (0.81-1.41)		1.01^g^ (0.76-1.34)	1.03 (0.58-1.84)		0.89^g^ (0.49-1.64)
	Regularly on-site	1.57 (1.30-1.89)***	1.47^f^ (1.19-1.80)***	1.50 (1.21-1.86)***		1.44^g^ (1.15-1.80)**	1.56 (1.03-2.36)*		1.25^g^ (0.78-2.00)
**Daily exposure risk trajectory**									
	Class 1	1.00	1.00	1.00		1.00	1.00		1.00
	Class 2	1.38 (1.08-1.77)*	1.38 (1.07-1.77)*	1.41 (1.07-1.84)*		1.33 (1.02-1.76)*	1.55 (0.78-3.10)		1.45 (0.72-2.90)
	Class 3	1.50 (1.18-1.90)**	1.49 (1.16-1.91)**	1.57 (1.21-2.04)***		1.46 (1.11-1.92)**	1.55 (0.78-3.05)		1.38 (0.69-2.76)
	Class 4	1.47 (1.09-1.99)*	1.46 (1.07-2.00)*	1.70 (1.23-2.36)**		1.55 (1.11-2.18)*	0.92 (0.39-2.18)		0.96 (0.40-2.27)
**Social exposure risk trajectory**									
	Class 1	1.00	1.00	1.00		1.00	1.00		1.00
	Class 2	0.99 (0.79-1.23)	1.04 (0.83-1.31)	1.01 (0.80-1.30)		1.02 (0.80-1.30)	1.06 (0.56-1.99)		1.11 (0.59-2.10)
	Class 3	1.07 (0.82-1.40)	1.16 (0.89-1.53)	1.10 (0.84-1.51)		1.11 (0.82-1.49)	1.20 (0.60-2.41)		1.23 (0.61-2.49)
	Class 4	1.35 (1.05-1.73)*	1.52 (1.18-1.97)**	1.40 (1.06-1.85)*		1.41 (1.06-1.87)*	1.66 (0.87-3.17)		1.91 (0.99-3.66)
	Class 5	1.03 (0.79-1.34)	1.19 (0.90-1.57)	1.01 (0.79-1.45)		1.14 (0.84-1.56)	1.13 (0.59-2.21)		1.42 (0.72-2.80)

^a^Adjusted for sex, age, education level, COVID-19 vaccination status, and work pattern. Cumulative incidence (as of December 2022)=51.6%.

^b^Adjusted for sex, age, education level, and work pattern. Cumulative incidence for pre-Omicron vaccination status: 0-2 doses=54.4%. Cumulative incidence of pre-Omicron vaccination status ≥3 doses=42.7%.

^c^HR: hazard ratio.

^d^aHR: adjusted hazard ratio.

^e^Not applicable

^f^Adjusted for sex, age, education level, and COVID-19 vaccination status.

^g^Adjusted for sex, age, and education level.

*P<.05; **P<.01; ***P<.001.

## Discussion

### Principal Findings

Based on the heterogeneous profiles of behavior changes over 3 epidemic waves, our results demonstrated the evolvement of the SARS-CoV-2 Omicron outbreak through different exposure settings unfolding in the workplace, accelerating in the household, and sustained in daily and social environments in an 11-month period. With similar travel restrictions and mandatory mask wearing in place during the whole study period, these patterns illuminate the apparently stereotyped evolution of exposure settings brought on by changes in social distancing interventions and population susceptibility. The enhanced understanding of the contextual variations along an epidemic trajectory could inform more targeted control strategies against future respiratory epidemics.

When the Omicron BA.2 lineage predominated in wave 1, individuals working outside the home, having more workplace contacts, and living in a larger household were at a higher risk of infection [19]. It can be deduced that, against a low baseline burden (<0.2%), the high population susceptibility had rendered a majority of virus transmissions initiated at the workplace and scattered to workers’ residences afterward. The office is a ubiquitous work setting in Hong Kong. Apart from health care facilities where outbreaks were commonly reported, the close proximity and prolonged contact between coworkers in the office could also create a favorable environment for virus transmission and contribute to the initial spread [20]. This could help explain the higher infection risk identified not merely in health care workers but also in the population not sheltered by remote work in our study [21]. In Hong Kong, WFH practice had never been mandated over the entire epidemic. The absence of linkage between workplace exposure risk and SARS-CoV-2 infection in later epidemic waves also suggested the effectiveness of alternative WFH interventions aside from a hard mandate (ie, forcing all employees to work from home). Alternatives such as assigning employees into cohorts to return to the workplace on alternate days and limiting the number of employees at any one time should be considered in future outbreaks to minimize societal costs [22].

Stemming from workplace outbreaks, household exposure to SARS-CoV-2 had probably culminated in early February 2022 when WFH interventions were introduced [23]. A similar transition of exposure setting was observed upon the emergence of COVID-19 elsewhere, proving that such a pattern was unlikely to be coincidental [24]. With extensive facility closures at tier 1, the halting of community activities had likely further compelled SARS-CoV-2 transmissions within the residence [25]. Such an epidemiological transition was mirrored in the outbreak dynamics, in which the precipitous rise mimicked the multiple infections established in fully susceptible households, whereas the exponential decline resulted from the limited onward transmission preempted by isolation [10]. In hindsight, discouraging multihousehold gatherings could be an effective means to confine the epidemic spread when new infections surge in the community [26]. The observed higher risk in larger households, however, did not corroborate other study findings [27,28]. A plausible explanation could be that the practice of home isolation, as a makeshift for the shortage of isolation facilities, had facilitated secondary transmission in larger households [29]. Being one of the most densely populated cities worldwide, the case in Hong Kong called for a strategic focus on the pragmatic approach of home quarantine and isolation in preparation for the emerging pandemics [30].

During wave 2 when Omicron BA.4/5 lineages prevailed, the outbreaks had most likely affected individuals with a higher daily exposure risk [31]. This pattern concurred with the partial reopening of community facilities at tier 2 and the results of another study [32]. A meta-analysis estimated a low attack rate for SARS-CoV-2 transmission derived from public and casual contacts [33]. The inefficient mode of spread, coupled with the accelerated third-dose vaccination uptake in previous months, had probably contributed to the milder outbreaks in wave 2 even when >70% remained uninfected in the population [34]. The findings also revealed that, although class 4 participants had a wider source of exposure through participation in more leisure activities than class 3 participants, the former was not significantly more vulnerable to infection. It is possible that the heightened perceived risk and precautionary behaviors compensated the actual risk [35]. Based on the subgroup analysis, a higher daily exposure risk did not result in an increased likelihood of SARS-CoV-2 acquisition among individuals having received ≥3 vaccine doses before the outbreak. This has somewhat suggested the effectiveness of booster vaccination in preventing infections arising from a lower infecting dose through casual exposure. Likewise, a previous study found that vaccinated health care workers before infection were more likely to report probable exposure in households than in other settings [36]. These findings supported in part the administration of vaccine passes as a means to allow the safe resumption of day-to-day activities in protected individuals in the face of similar epidemics [37].

With presumably >30% of the population conferred with hybrid immunity resulting from the 2 epidemic waves, the number of incident cases in wave 3 had somewhat exceeded expectations in the lack of reinfection cases [38]. It is hypothesized that following tier 4 relaxation, the reinstatement of a larger gathering had rendered an increased virus exposure to socially active subgroups such as social exposure risk trajectory class 3 and class 5 participants, who might have played a part in extending the outbreaks. Although we could not rule out that the imported traces of the XBB lineage had aided in the pervasive spread [39], the phenomenon resembled previous findings revealing a higher SARS-CoV-2 susceptibility in individuals who attended meetings of ≥10 people [40] and celebrative events [41]. The observation should, however, be interpreted with caution since exposure to a virus in a social context necessitates the presence of an infected seed in one’s social network [42]. Of note, older individuals presented a higher social exposure risk. This could partly be due to the uninterrupted practice of yum-cha (dining and socializing at a dim sum restaurant) when eateries remained open throughout the epidemic [43]. Furthermore, male participants were less likely to adjust their social behaviors along the progression of the epidemic. These findings convey the need to promote compliance by targeting specific community groups differentiated by age and gender in future epidemics [44].

### Limitations in This Study

Several limitations existed in this study. The self-reported nature of SARS-CoV-2 infection could lead to an underestimation of the cumulative incidence if infected participants did not respond to the follow-up survey in that month. Such bias was minimized by excluding participants without adequate follow-up responses from analyses. The higher vaccination uptake in older individuals deviating from reality also informed the presence of a selection bias. This could be subject to the recruitment of older adults who have a higher computer literacy, which enabled them to acquire more information about COVID-19 vaccination online [45]. Recall bias could also be generated from the retrospective report of activities. This was minimized by restricting the accessibility of the follow-up survey to the first 14 days of each month. Social desirability bias could also be present when participants failed to disclose activities, which were against the quarantine and isolation orders. Furthermore, the role of virus transmission in school was not captured, despite its effect being anticipated to be small, since normal schooling was not resumed in most parts of the study period. The timing of survey initiation also coincided with the inception of the Omicron outbreak. The absence of pre-Omicron data impeded our inference on the change in the exposure risk pattern compared to the baseline. The results should also be interpreted with caution in light of the different transmissibility between the Omicron lineages [46]. The impact was, however, assumed to be small in a highly vaccinated population, as the effectiveness of mRNA vaccines against Omicron BA.2 did not differ significantly compared to BA.5 infection [47].

### Conclusions

Monitoring the shift of exposure settings in future epidemics is important, as the pattern could inform the effective calibration of social distancing measures in a targeted manner as opposed to an exhaustive lockdown. When formulating preparedness and response plans for emerging epidemics of respiratory viruses with similar transmission dynamics, policy makers should also take such contextual variations and the accompanying effects into consideration. In a community-wide outbreak, where the workplace happens to be the first epicenter, health authorities should act in anticipation of the rise in intrahousehold transmission when WFH interventions are expected. Later strategies could focus on the prevention of explosive outbreaks in pursuit of maximum resumption of daily and social activities in protected individuals. In light of the identified patterns, future research may also explore the broader implications of exposure settings on other NPIs such as the effectiveness of masking. Methodologically, this study demonstrates the capability of a participatory surveillance cohort in examining the effect of social distancing measures on population exposure and disease patterns. This has introduced opportunities for its wider applications in detecting early epidemiological patterns and retrospectively evaluating epidemic responses for future outbreaks.

## Appendix

Multimedia Appendix 1 Supplementary tables and figures.

## Abbreviations

aHR: adjusted hazard ratio
aOR: adjusted odds ratio
BIC: Bayesian information criterion
HR: hazard ratio
NPI: nonpharmaceutical intervention
RR: relative risk
WFH: work from home

## Data Availability

The questionnaire data are protected, as consent for sharing individual-level information was not obtained from research participants. The source data for each figure in aggregate form are available upon reasonable request from the corresponding author.

## References

[1] (2020). COVID-19 Thematic Website - Together, We Fight the Virus.

[2] Menegale F, Manica M, Zardini A, Guzzetta G, Marziano V, d'Andrea V, Trentini F, Ajelli M, Poletti P, Merler S (2023). Evaluation of waning of SARS-CoV-2 vaccine-induced immunity: a systematic review and meta-analysis. JAMA Netw Open.

[3] Jarvis CI, Van Zandvoort K, Gimma A, Prem K, Klepac Petra, Rubin G James, Edmunds W John, CMMID COVID-19 working group (2020). Quantifying the impact of physical distance measures on the transmission of COVID-19 in the UK. BMC Med.

[4] Tomori DV, Rübsamen Nicole, Berger T, Scholz S, Walde J, Wittenberg I, Lange B, Kuhlmann A, Horn J, Mikolajczyk R, Jaeger VK, Karch A (2021). Individual social contact data and population mobility data as early markers of SARS-CoV-2 transmission dynamics during the first wave in Germany-an analysis based on the COVIMOD study. BMC Med.

[5] Coleman N, Gao X, DeLeon J, Mostafavi A (2022). Human activity and mobility data reveal disparities in exposure risk reduction indicators among socially vulnerable populations during COVID-19 for five U.S. metropolitan cities. Sci Rep.

[6] Whittemore K, Foerster S, Blaney K, Long T, Vora NM (2022). Evaluation of risk factors for conversion from a COVID-19 household contact to a case in New York City, August 1, 2020, to July 31, 2021. JAMA Netw Open.

[7] Shah VP, Breeher LE, Alleckson JM, Rivers DG, Wang Z, Stratton ER, Farah W, Hainy CM, Swift MD (2022). Occupational exposure to severe acute respiratory coronavirus virus 2 (SARS-CoV-2) and risk of infection among healthcare personnel. Infect Control Hosp Epidemiol.

[8] Zhao Y, Zhao S, Guo Z, Yuan Z, Ran J, Wu L, Yu L, Li H, Shi Y, He D (2022). Differences in the superspreading potentials of COVID-19 across contact settings. BMC Infect Dis.

[9] Wong NS, Lee SS, Kwan TH, Yeoh E (2020). Settings of virus exposure and their implications in the propagation of transmission networks in a COVID-19 outbreak. Lancet Reg Health West Pac.

[10] Imamura T, Watanabe A, Serizawa Y, Nakashita M, Saito M, Okada M, Ogawa A, Tabei Y, Soumura Y, Nadaoka Y, Nakatsubo N, Chiba T, Sadamasu K, Yoshimura K, Noda Y, Iwashita Y, Ishimaru Y, Seki N, Otani K, Imamura T, Griffith MM, DeToy K, Suzuki M, Yoshida M, Tanaka A, Yauchi M, Shimada T, Oshitani H (2023). Transmission of COVID-19 in nightlife, household, and health care settings in Tokyo, Japan, in 2020. JAMA Netw Open.

[11] Kwan TH, Wong NS, Yeoh E, Lee SS (2023). Shifts of SARS-CoV-2 exposure settings in the transmission clusters of 2 epidemic waves in Hong Kong. Int J Environ Health Res.

[12] Akhmetzhanov AR, Cheng H, Linton NM, Ponce L, Jian S, Lin H (2022). Transmission dynamics and effectiveness of control measures during COVID-19 surge, Taiwan, April-August 2021. Emerg Infect Dis.

[13] Wong K, Gimma A, Coletti P, Faes Christel, Beutels Philippe, Hens Niel, Jaeger Veronika K, Karch Andre, Johnson Helen, Edmunds WJohn, Jarvis Christopher I, CoMix Europe Working Group (2023). Social contact patterns during the COVID-19 pandemic in 21 European countries - evidence from a two-year study. BMC Infect Dis.

[14] Hong B, Bonczak BJ, Gupta A, Thorpe LE, Kontokosta CE (2021). Exposure density and neighborhood disparities in COVID-19 infection risk. Proc Natl Acad Sci U S A.

[15] Wittwer S, Paolotti D, Lichand G, Leal Neto O (2023). Participatory surveillance for COVID-19 trend detection in Brazil: cross-sectional study. JMIR Public Health Surveill.

[16] Koppeschaar CE, Colizza V, Guerrisi C, Turbelin C, Duggan J, Edmunds WJ, Kjelsø Charlotte, Mexia R, Moreno Y, Meloni S, Paolotti D, Perrotta D, van Straten E, Franco AO (2017). Influenzanet: citizens among 10 countries collaborating to monitor influenza in Europe. JMIR Public Health Surveill.

[17] (2022). 2021 Population Census Internet.

[18] Jung T, Wickrama KAS (2007). An introduction to latent class growth analysis and growth mixture modeling. Soc Pers Psych.

[19] Xie R, Edwards KM, Adam DC, Leung KSM, Tsang TK, Gurung S, Xiong W, Wei X, Ng DYM, Liu GYZ, Krishnan P, Chang LDJ, Cheng SMS, Gu H, Siu GKH, Wu JT, Leung GM, Peiris M, Cowling BJ, Poon LLM, Dhanasekaran V (2023). Resurgence of Omicron BA.2 in SARS-CoV-2 infection-naive Hong Kong. Nat Commun.

[20] Oude Hengel KM, Burdorf A, Pronk A, Schlünssen Vivi, Stokholm ZA, Kolstad HA, van Veldhoven K, Basinas I, van Tongeren M, Peters S (2022). Exposure to a SARS-CoV-2 infection at work: development of an international job exposure matrix (COVID-19-JEM). Scand J Work Environ Health.

[21] Luckhaupt SE, Horter L, Groenewold MR, de Perio MA, Robbins CL, Sweeney MH, Thomas I, Valencia D, Ingram A, Heinzerling A, Nguyen A, Townsend EB, Weber RC, Reichbind D, Dishman H, Kerins JL, Lendacki FR, Austin C, Dixon L, Spillman B, Simonson S, Tonzel J, Krueger A, Duwell M, Bachaus B, Rust B, Barrett C, Morrison B, Owers Bonner KA, Karlsson ND, Angelon-Gaetz K, McClure ES, Kline KE, Dangar D, Reed C, Karpowicz J, Anderson SM, Cantor S, Chaudhary I, Ellis EM, Taylor ML, Sedon A, Kocharian A, Morris C, Samson ME, Mangla AT (2023). COVID-19 outbreaks linked to workplaces, 23 US jurisdictions, August-October 2021. Public Health Rep.

[22] Beale S, Hoskins S, Byrne T, Fong WLE, Fragaszy E, Geismar C, Kovar J, Navaratnam AM, Nguyen V, Patel P, Yavlinsky A, Johnson AM, Van Tongeren M, Aldridge RW, Hayward A (2022). Workplace contact patterns in England during the COVID-19 pandemic: analysis of the Virus Watch prospective cohort study. Lancet Reg Health Eur.

[23] (2022). Government enhances anti-epidemic measures in view of severe epidemic situation. The Government of the Hong Kong Special Administrative Region.

[24] Murti M, Achonu C, Smith B, Brown K, Kim J, Johnson J, Ravindran S, Buchan S (2021). COVID-19 workplace outbreaks by industry sector and their associated household transmission, Ontario, Canada, January to June, 2020. J Occup Environ Med.

[25] Trentini F, Manna A, Balbo N, Marziano V, Guzzetta G, O'Dell Samantha, Kummer AG, Litvinova M, Merler S, Ajelli M, Poletti P, Melegaro A (2022). Investigating the relationship between interventions, contact patterns, and SARS-CoV-2 transmissibility. Epidemics.

[26] Schepers M, Zanger P, Jahn K, König Jochem, Strauch K, Gianicolo E (2022). Multi-household social gatherings contribute to the second SARS-CoV-2 wave in Rhineland-Palatinate, Germany, August to November 2020. J Infect.

[27] Islam F, Alvi Y, Ahmad M, Ahmed F, Rahman A, Singh FHD, Das AK, Dudeja M, Gupta E, Agarwalla R, Alam I, Roy S (2023). Household transmission dynamics of COVID-19 among residents of Delhi, India: a prospective case-ascertained study. IJID Reg.

[28] Lopez Bernal Jamie, Panagiotopoulos N, Byers C, Garcia Vilaplana Tatiana, Boddington N, Zhang X, Charlett Andre, Elgohari Suzanne, Coughlan Laura, Whillock Rosie, Logan Sophie, Bolt Hikaru, Sinnathamby Mary, Letley Louise, MacDonald Pauline, Vivancos Roberto, Edeghere Obaghe, Anderson Charlotte, Paranthaman Karthik, Cottrell Simon, McMenamin Jim, Zambon Maria, Dabrera Gavin, Ramsay Mary, Saliba Vanessa (2022). Transmission dynamics of COVID-19 in household and community settings in the United Kingdom, January to March 2020. Euro Surveill.

[29] Paul L, Daneman N, Brown K, Johnson J, van Ingen Trevor, Joh E, Wilson Sarah E, Buchan Sarah A (2021). Characteristics associated with household transmission of severe acute respiratory syndrome coronavirus 2 (SARS-CoV-2) in Ontario, Canada: a cohort study. Clin Infect Dis.

[30] Wong NS, Lee KCK, Kwan TH, Lee SS (2021). Practicalities of home quarantine for city dwellers with limited living space. Asia Pac J Public Health.

[31] Guo Z, Zhao S, Yam CHK, Li C, Jiang X, Chow TY, Chong KC, Yeoh EK (2023). Estimating the serial intervals of SARS-CoV-2 Omicron BA.4, BA.5, and BA.2.12.1 variants in Hong Kong. Influenza Other Respir Viruses.

[32] Manout O, Ciari F (2021). Assessing the role of daily activities and mobility in the spread of COVID-19 in Montreal with an agent-based approach. Front Built Environ.

[33] Thompson H, Mousa A, Dighe A, Fu H, Arnedo-Pena A, Barrett P, Bellido-Blasco Juan, Bi Qifang, Caputi Antonio, Chaw Liling, De Maria Luigi, Hoffmann Matthias, Mahapure Kiran, Ng Kangqi, Raghuram Jagadesan, Singh Gurpreet, Soman Biju, Soriano Vicente, Valent Francesca, Vimercati Luigi, Wee Liang En, Wong Justin, Ghani Azra C, Ferguson Neil M (2021). Severe acute respiratory syndrome coronavirus 2 (SARS-CoV-2) setting-specific transmission rates: a systematic review and meta-analysis. Clin Infect Dis.

[34] Zhou R, Liu N, Li X, Peng Q, Yiu C, Huang H, Yang D, Du Z, Kwok H, Au K, Cai J, Fan-Ngai Hung I, Kai-Wang To K, Xu X, Yuen K, Chen Z (2023). Three-dose vaccination-induced immune responses protect against SARS-CoV-2 Omicron BA.2: a population-based study in Hong Kong. Lancet Reg Health West Pac.

[35] Wang R, Zhang Y, Wu R, Li B, Li C, Yu B, Zhang Y (2021). A study of self-precaution against the background of the COVID-19 pandemic from the perspective of risk perception attitude theory and social support. BMC Public Health.

[36] Oster Y, Benenson S, Yochi Harpaz L, Buda I, Nir-Paz R, Strahilevitz J, Cohen MJ (2021). Association between exposure characteristics and the risk for COVID-19 infection among health care workers with and without BNT162b2 vaccination. JAMA Netw Open.

[37] Sharif A, Botlero R, Hoque N, Alif SM, Nazmul Karim M, Islam SMS (2021). A pragmatic approach to COVID-19 vaccine passport. BMJ Glob Health.

[38] Huang L, Lai FTT, Yan VKC, Cheng FWT, Cheung CL, Chui CSL, Li X, Wan EYF, Wong CKH, Hung IFN, Lau CS, Wong ICK, Chan EWY (2022). Comparing hybrid and regular COVID-19 vaccine-induced immunity against the Omicron epidemic. NPJ Vaccines.

[39] (2023). Latest situation of COVID-19 (as of 1 January 2023). Centre for Health Protection.

[40] Lin A, Vittinghoff E, Olgin J, Peyser N, Aung S, Joyce S, Yang V, Hwang J, Avram R, Nah G, Tison GH, Beatty A, Runge R, Wen D, Butcher X, Horner C, Eitel H, Pletcher M, Marcus GM (2021). Predictors of incident SARS-CoV-2 infections in an international prospective cohort study. BMJ Open.

[41] Whaley CM, Cantor J, Pera M, Jena AB (2021). Assessing the association between social gatherings and COVID-19 risk using birthdays. JAMA Intern Med.

[42] Volz EM, Miller JC, Galvani A, Ancel Meyers L (2011). Effects of heterogeneous and clustered contact patterns on infectious disease dynamics. PLoS Comput Biol.

[43] (2022). No jab, no dim sum: how food got Hong Kong's elderly vaccinated. South China Morning Post.

[44] Pflugeisen BM, Mou J (2021). Gender discrepancies in SARS-CoV-2 pandemic related beliefs, attitudes, and practices. Front Public Health.

[45] Zhang D, Zhou W, Poon PK, Kwok KO, Chui TW, Hung PHY, Ting BYT, Chan DC, Wong SY (2022). Vaccine resistance and hesitancy among older adults who live alone or only with an older partner in community in the early stage of the fifth wave of COVID-19 in Hong Kong. Vaccines (Basel).

[46] Shrestha LB, Foster C, Rawlinson W, Tedla N, Bull RA (2022). Evolution of the SARS-CoV-2 omicron variants BA.1 to BA.5: implications for immune escape and transmission. Rev Med Virol.

[47] Kislaya I, Casaca P, Borges V, Sousa C, Ferreira BI, Fonte A, Fernandes E, Dias CM, Duarte S, Almeida JP, Grenho I, Coelho L, Ferreira R, Ferreira PP, Borges CM, Isidro J, Pinto M, Menezes L, Sobral D, Nunes A, Santos D, Gonçalves António Maia, Vieira L, Gomes JP, Leite PP, Nunes B, Machado A, Peralta-Santos A (2023). Comparative effectiveness of COVID-19 vaccines in preventing infections and disease progression from SARS-CoV-2 Omicron BA.5 and BA.2, Portugal. Emerg Infect Dis.

