# Supplementary material for: Population Behavior Changes Underlying Phasic Shifts of SARS-CoV-2 Exposure Settings Across 3 Omicron Epidemic Waves in Hong Kong: Prospective Cohort Study
Source: JMIR Public Health Surveill. 2024 Jun 19;10:e51498. doi: 10.2196/51498 (PMC11222765; doi:10.2196/51498)

**Table S1. Distribution of the recruited households by 18 districts**

|  | **Recruited households** | | **2021 Population Census** | |  |
| --- | --- | --- | --- | --- | --- |
| **Districts** | **n** | **%** | **n** | **%** | **Difference** |
| Central and Western | 169 | 3.2% | 88922 | 3.3% | -0.1% |
| Eastern | 407 | 7.6% | 188706 | 7.1% | +0.6% |
| Islands | 116 | 2.2% | 67104 | 2.5% | -0.3% |
| Kowloon City | 313 | 5.9% | 147023 | 5.5% | +0.4% |
| Kwai Tsing | 343 | 6.4% | 176604 | 6.6% | -0.2% |
| Kwun Tong | 500 | 9.4% | 247592 | 9.3% | +0.1% |
| North | 217 | 4.1% | 111433 | 4.2% | -0.1% |
| Sai Kung | 353 | 6.6% | 167771 | 6.3% | +0.4% |
| Sham Shui Po | 302 | 5.7% | 165439 | 6.2% | -0.5% |
| Shatin | 469 | 8.8% | 246425 | 9.2% | -0.4% |
| Southern | 173 | 3.3% | 86479 | 3.2% | +0.0% |
| Tai Po | 193 | 3.6% | 110747 | 4.1% | -0.5% |
| Tsuen Wan | 279 | 5.2% | 114009 | 4.3% | +1.0% |
| Tuen Mun | 397 | 7.5% | 187202 | 7.0% | +0.5% |
| Wanchai | 116 | 2.2% | 63128 | 2.4% | -0.2% |
| Wong Tai Sin | 284 | 5.3% | 147085 | 5.5% | -0.2% |
| Yau Tsim Mong | 232 | 4.4% | 123707 | 4.6% | -0.3% |
| Yuen Long | 458 | 8.6% | 234338 | 8.8% | -0.2% |
| **Total** | **5321** | **100%** | **2673714** | **100%** | **--** |

**Table S2. Comparison of characteristics between participants included in and excluded from the latent class growth model and analyses thereafter**

| **Characteristics** | **Included  (n=1240)** | **Excluded (n=4081)** | ***P ^a^*** |
| --- | --- | --- | --- |
| **Sex** |  |  |  |
| Male | 515 (41.5) | 1706 (41.8) | .89 |
| Female | 725 (58.5) | 2375 (58.2) |  |
| **Age (median, interquartile range)** | 48 (37-58) | 45 (34-57) | <.001 |
| 18-29 years | 130 (10.5) | 602 (14.8) | <.001 |
| 30-39 years | 261 (21.0) | 927 (22.7) |  |
| 40-49 years | 267 (21.5) | 913 (22.4) |  |
| 50-64 years | 442 (35.6) | 1172 (28.7) |  |
| ≥ 65 years | 140 (11.3) | 467 (11.4) |  |
| **Ethnicity** |  |  |  |
| Local (Hong Kong) Chinese | 1222 (98.5) | 3968 (97.2) | .012 |
| Non-local Chinese/other ethnicities | 18 (1.5) | 113 (2.8) |  |
| **Education level** |  |  |  |
| Primary education or below | 30 (2.4) | 145 (3.6) | .07 |
| Secondary education | 403 (32.5) | 1418 (34.7) |  |
| Diploma/associate degree | 190 (15.3) | 613 (15.0) |  |
| Bachelor’s degree or above | 617 (49.8) | 1905 (46.7) |  |
| **Employment status** |  |  |  |
| Full-time employment/self-employed | 760 (61.3) | 2448 (60) | .41 |
| Part-time/temporary employment | 59 (4.8) | 229 (5.6) |  |
| Student | 47 (3.8) | 204 (5.0) |  |
| Homemaker | 94 (7.6) | 312 (7.6) |  |
| Unemployed | 81 (6.5) | 269 (6.6) |  |
| Retired | 199 (16) | 617 (15.1) |  |
| **Monthly income range (n = 3739)** | (missing = 1) | (missing = 7) |  |
| < HK$10,000 | 95 (11.0) | 339 (11.8) | .58 |
| HK$10,000 – 19,999 | 211 (24.4) | 684 (23.8) |  |
| HK$20,000 – 29,999 | 184 (21.3) | 575 (20.0) |  |
| HK$30,000 – 59,999 | 213 (24.6) | 759 (26.4) |  |
| ≥ HK$60,000 | 105 (12.1) | 305 (10.6) |  |
| Refuse to answer | 57 (6.6) | 212 (7.4) |  |
| **Reported chronic illness ^b^** | (unsure = 56) | (unsure = 190) |  |
| No | 829 (70.0) | 2809 (72.2) | .16 |
| Yes | 355 (30.0) | 1082 (27.8) |  |
| **Household size** | (missing = 22) | (missing = 40) |  |
| Mean, standard deviation | 3.07 (1.41) | 3.14 (1.49) | .16 |
| 1 (living alone) | 139 (11.4) | 468 (11.6) | .22 |
| 2 persons | 350 (28.7) | 1128 (27.9) |  |
| 3-4 persons | 542 (44.5) | 1722 (42.6) |  |
| 5-6 persons | 170 (14.0) | 635 (15.7) |  |
| ≥ 7 persons | 17 (1.4) | 88 (2.2) |  |

a. Based on Chi-square test for categorical variables and Mann-Whitney U test for continuous variables

b. Including any diagnosed conditions which require long-term clinical follow-up or medications, such as hypertension, diabetes mellitus, stroke, asthma etc.

**Table S2. Fit indices and predicted class membership for the latent class growth models of work pattern and exposure risk dynamics in daily and social setting**

**(A) Work pattern**

| **Number of**  **latent class** | **Fit indices** | | | **Predicted class membership (posterior probabilities)** | | | | | | |
| --- | --- | --- | --- | --- | --- | --- | --- | --- | --- | --- |
|  | **BIC** | **ssBIC** | **Entropy** | **Class 1, %** | **Class 2, %** | **Class 3, %** | **Class 4, %** | **Class 5, %** | **Class 6, %** | **Class 7, %** |
| 1 class | 22793.77 | 22781.07 | -- | 100 (100) | -- | -- | -- | -- | -- | -- |
| 2 classes | 14255.97 | 14230.56 | 0.986 | 32.3 (99.5) | 67.7 (99.7) | -- | -- | -- | -- | -- |
| 3 classes | 12308.19 | 12270.07 | 0.957 | 28.7 (99.9) | 19.1 (94.6) | 52.2 (98.4) | -- | -- | -- | -- |
| 4 classes | 12039.89 | 11989.07 | 0.901 | 16.9 (95.4) | 28.7 (99.9) | 32.7 (95.5) | 21.7 (82.7) | -- | -- | -- |
| 5 classes | 11866.07 | 11802.54 | 0.865 | 22.2 (82.4) | 28.7 (99.8) | 22.6 (91.7) | 12.4 (87.3) | 14.1 (93.1) | -- | -- |
| 6 classes | 11745.30 | 11669.07 | 0.881 | 22.0 (83.6) | 1.9 (90.2) | 22.3 (91.7) | 12.0 (87.7) | 28.7 (99.9) | 13.1 (93.0) | -- |
| 7 classes | 11659.33 | 11570.39 | 0.887 | 1.9 (90.3) | 11.5 (89.2) | 2.2 (88.7) | 21.8 (84.1) | 28.7 (99.8) | 22.2 (90.7) | 11.8 (92.1) |

**(B) Daily exposure risk**

| **Number of**  **latent class** | **Fit indices** | | | **Predicted class membership (posterior probabilities)** | | | | | | |
| --- | --- | --- | --- | --- | --- | --- | --- | --- | --- | --- |
|  | **BIC** | **ssBIC** | **Entropy** | **Class 1, %** | **Class 2, %** | **Class 3, %** | **Class 4, %** | **Class 5, %** | **Class 6, %** | **Class 7, %** |
| 1 class | 30156.06 | 30140.17 | -- | 100 (100) | -- | -- | -- | -- | -- | -- |
| 2 classes | 26887.10 | 26858.51 | 0.862 | 46.0 (95.7) | 54.0 (96.2) | -- | -- | -- | -- | -- |
| 3 classes | 26072.05 | 26030.75 | 0.852 | 17.3 (92.7) | 51.7 (92.9) | 31.1 (94.7) | -- | -- | -- | -- |
| 4 classes | 25766.03 | 25712.03 | 0.801 | 17.6 (91.1) | 32.9 (86.2) | 38.1 (88.1) | 11.5 (90.5) | -- | -- | -- |
| 5 classes | 25703.68 | 25636.97 | 0.754 | 15.6 (75.0) | 38.1 (86.9) | 19.0 (76.1) | 16.2 (90.6) | 11.0 (89.8) | -- | -- |
| 6 classes | 25654.18 | 25574.77 | 0.751 | 16.5 (90.5) | 10.4 (89.7) | 15.6 (73.4) | 19.1 (76.0) | 4.5 (74.2) | 33.9 (84.2) | -- |
| 7 classes | 25648.62 | 25556.50 | 0.730 | 10.6 (84.4) | 11.2 (69.) | 10.2 (89.2) | 15.3 (74.3) | 14.8 (72.0) | 4.2 (74.0) | 33.7 (83.9) |

**(C) Social exposure risk**

| **Number of**  **latent class** | **Fit indices** | | | **Predicted class membership (posterior probabilities)** | | | | | | |
| --- | --- | --- | --- | --- | --- | --- | --- | --- | --- | --- |
|  | **BIC** | **ssBIC** | **Entropy** | **Class 1, %** | **Class 2, %** | **Class 3, %** | **Class 4, %** | **Class 5, %** | **Class 6, %** | **Class 7, %** |
| 1 class | 31859.77 | 31843.89 | -- | 100 (100) | -- | -- | -- | -- | -- | -- |
| 2 classes | 27711.51 | 27682.92 | 0.878 | 51.9 (96.0) | 48.1 (96.7) | -- | -- | -- | -- | -- |
| 3 classes | 26092.46 | 26051.17 | 0.889 | 26.1 (95.0) | 49.7 (94.0) | 24.2 (96.2) | -- | -- | -- | -- |
| 4 classes | 25674.03 | 25620.03 | 0.842 | 21.0 (94.4) | 15.2 (92.8) | 35.3 (90.2) | 28.5 (88.2) | -- | -- | -- |
| 5 classes | 25503.30 | 25436.59 | 0.816 | 21.0 (94.5) | 33.1 (90.1) | 14.6 (78.6) | 16.7 (81.0) | 14.6 (92.6) | -- | -- |
| 6 classes | 25429.78 | 25350.36 | 0.768 | 13.3 (91.1) | 14.5 (81.2) | 19.0 (77.5) | 17.5 (78.8) | 17.3 (78.5) | 18.4 (92.5) | -- |
| 7 classes | 25405.37 | 25313.25 | 0.743 | 14.0 (80.1) | 16.5 (74.5) | 16.2 (77.4) | 14.3 (80.2) | 10.2 (85.1) | 13.2 (91.3) | 15.6 (73.4) |

BIC = Bayesian Information Criterion; ssBIC = Sample size-adjusted Bayesian Information Criterion

**Figure S1. Daily incident SARS-CoV-2 cases and the four tiers of social distancing policy in Hong Kong from February to December 2022**

**Tier 2 (21/4 – 18/5)**

**Eatery**: 4 persons; closing at 10pm

**Banquet**: 20 persons

**Bar/pub**: closed

**Scheduled premises^1^**: PARTIALLY reopened

**Multi-household gathering**: allowed

**Local tour**: allowed at a capacity of 30/100 persons

**Work-from-home^2^:** no

**Mask mandate:** yes

**Tier 4 (6/10 – 21/12)**

**Eatery**: 12 persons; closing at 12mn

**Banquet**: 240 persons

**Bar/pub**: 6 persons; closing at 2am

**Scheduled premises^1^**: FULLY reopened

**Multi-household gathering**: allowed

**Local tour**: allowed at a capacity of 30/100 persons

**Work-from-home^2^:** no

**Mask mandate:** yes

**Tier 3 (19/5 – 5/10)**

**Eatery**: 8 persons; closing at 10pm

**Banquet**: 120 persons

**Bar/pub**: 4 persons; closing at 2am

**Scheduled premises^1^:** FULLY reopened

**Multi-household gathering**: allowed

**Local tour**: allowed at a capacity of 30/100 persons

**Work-from-home^2^:** no

**Mask mandate:** yes

**Tier 1 (10/2 – 20/4)**

**Eatery**: 2 persons; closing at 6pm

**Banquet**: prohibited

**Bar/pub:** closed

**Scheduled premises^1^:** ALL closed

**Multi-household gathering:** prohibited

**Local tour:** prohibited

**Work-from-home^2^:** yes

**Mask mandate:** yes

**Feb Mar Apr May Jun Jul Aug Sep Oct Nov Dec**

**Presentation of a negative RAT result for SARS-CoV-2 upon entry to bar, pub, nightclub, and attendance of banquet (16/6 – 21/12)**

**1 dose (20/3) 2 doses (30/4) 3 doses (31/5)**

**Vaccine Pass^3^: minimum dose requirement for entry to specific premises**

4

2

3

1

^1^ Scheduled premises included but not limited to bathhouse, fitness centre, place of amusement and public entertainment, party room, beauty parlour, club-house, nightclub, karaoke establishment, mahjong-tin kau premises, massage establishment, sports premises, swimming pool/beach, religious premises

^2^ Based on the recommended work arrangement for government employees

^3^ Waived for individuals with SARS-CoV-2 infection reported within 6 months

**Figure S2. Elbow plot for latent class growth model of work pattern**


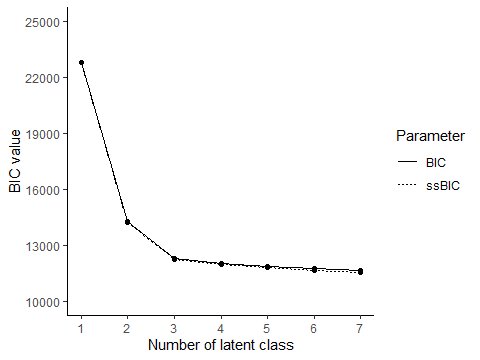

Supplement: Multimedia Appendix 1 [file publichealth_v10i1e51498_app1.docx]
